# “I have to live like I’m old.” Young adults’ perspectives on managing hypertension: a multi-center qualitative study

**DOI:** 10.1186/s12875-016-0428-9

**Published:** 2016-03-11

**Authors:** Heather M. Johnson, Ryan C. Warner, Jamie N. LaMantia, Barbara J. Bowers

**Affiliations:** Department of Medicine, School of Medicine and Public Health, University of Wisconsin, H4/512 CSC, MC 3248, 600 Highland Avenue, Madison, WI 53792 USA; Health Innovation Program, University of Wisconsin School of Medicine and Public Health, 800 University Bay Drive, Suite 210, Box 9445, Madison, WI 53705 USA; Department of Counselor Education and Counseling Psychology, Marquette University, Schroeder Health & Education Complex, 561 N 15th Street, Room 151A, Milwaukee, WI 53233 USA; Department of Research, School of Nursing, University of Wisconsin, 5130 Cooper Hall, Signe Skott, 701 Highland Avenue, Madison, WI 53705 USA; Department of Academic & Student Services, School of Nursing, University of Wisconsin, Cooper Hall, Suite 1100, 701 Highland Avenue, Madison, WI 53705 USA; Division of Cardiovascular Medicine, University of Wisconsin-Madison, School of Medicine and Public Health, UW Health Advanced Hypertension Program, H4/512 CSC, MC 3248, 600 Highland Avenue, Madison, WI 53792 USA

**Keywords:** Qualitative research, Hypertension, Ambulatory care, Health behavior, Young adults, Primary healthcare

## Abstract

**Background:**

In the U.S., young adults (18–39 year-olds) have the lowest hypertension control rates among hypertensive adults. Understanding young adults’ unique perceptions about hypertension and perceived barriers to hypertension control is critical to develop effective interventions for this population. This multi-center study explored young adults’: 1) emotions and reactions after a hypertension diagnosis, 2) attitudes about managing hypertension (lifestyle changes, follow-up visits, antihypertensive medication use), 3) opinions about their healthcare system’s hypertension education materials, and 4) opinions about using social media to manage hypertension.

**Methods:**

Young adults (18–39 year-olds) with a diagnosis of hypertension and regular primary care access were recruited by the Wisconsin Research and Education Network (WREN). Two focus groups (one per age range: 18–29 years, 30–39 years) were conducted in three Midwestern Family Medicine Clinics (academic, rural, and urban). Conventional content analysis was performed.

**Results:**

Thirty-eight young adults (mean: 26.7 [9.6] years old, 34 % male, 45 % Black, 42 % with ≥1 year of college) identified barriers to managing hypertension. Emergent themes overlapped across age groups and geographic regions. Most respondents were surprised and angry about a hypertension diagnosis; they expected to develop hypertension, but at a much older age. A hypertension diagnosis negatively altered their “young” self-identity; suggested behavior changes and antihypertensive medications made them feel “older” than their peers. Young adults missed blood pressure follow-up visits due to co-payments, transportation barriers, and longer than desired wait times for brief visits. Contrary to our hypothesis, most young adults disliked social media or text messaging to support self-management; they were most concerned that their peers would see the hypertension communication. Current hypertension education materials were described as not addressing young adults’ health questions and are often discarded before leaving the clinic.

**Conclusions:**

Targeting interventions to young adults’ unique needs is necessary to improve hypertension control and cardiovascular preventive healthcare delivery.

## Background

Uncontrolled hypertension in young adults (18–39 year-olds) is an enormous public health burden [1, 2]. In the U.S., approximately 1 in 5 young men and 1 in 6 young women have hypertension [3–6], increasing their risk of future heart failure, stroke, and chronic kidney disease [3, 7–10]. Given their longer exposure to high blood pressures, young adults with hypertension have a higher lifetime risk for cardiovascular disease [10–16].

Multiple prior studies have assessed barriers to hypertension control [17–20], highlighting patient, provider, and healthcare system factors [21–23]. Our previous research demonstrated important provider barriers in the management of young adults with incident (new) hypertension, including low rates of documented lifestyle counseling [24] and significant delays prescribing initial antihypertensive medication [25]. However, to identify barriers to hypertension control specific to young adults and develop effective interventions, we need insight from young adults about their experiences. Prior cross-cultural qualitative research by Morgan and Watkins focused on barriers to hypertension control of 35–55 year-olds and primarily on antihypertensive therapy [26]. However, the National Health and Nutrition Examination Survey (NHANES) demonstrated that 18–39 year-olds have the lowest hypertension control rates. Only 35 % of young adults with hypertension in the U.S. have achieved hypertension control (blood pressure <140/90 mmHg) [27, 28] compared to middle-aged (40–59 year-olds; 58 %) and older adults (≥60 year-olds; 54 %) [3]. In addition, a trial of lifestyle modifications is commonly the initial hypertension treatment step, rather than antihypertensive medication, among young adults [25]. An analysis by Savoca, et al. among African-American young adults demonstrated limited knowledge on risks of developing hypertension, and highlighted the need for intensive young adult education interventions [29]. Additionally, an extensive multi-ethnic systematic review of hypertension qualitative studies from 16 countries focused on hypertension treatment adherence [30]. Across ethnicities, patients reported disliking hypertension treatment, intentionally stopping treatment, and identified barriers to hypertension treatment (finance, time, memory) [30]. However, the authors concluded that to improve hypertension interventions, a better understanding of patients’ experiences is needed. Therefore, we conducted a qualitative study of young adults with hypertension, across multiple Midwest healthcare systems, to assess their perspective of barriers to hypertension treatment and control. In a separate manuscript, we analyzed qualitative data from primary care providers.

## Methods

### Young adult focus groups

This study was approved by the University of Wisconsin-Madison Health Sciences Institutional Review Board. Focus groups of young adults with hypertension were conducted at three Family Medicine clinics within three different counties in Wisconsin, including an academic community clinic, urban clinic, and rural clinic. Focus groups were the selected methodology, rather than individual interviews, in order to maximize interactions between the young adult participants. Our goal was to have young adults discuss experiences and barriers to managing hypertension, and brainstorm possible solutions to improve hypertension control and healthcare delivery for their age group. Interactions between participants are a unique feature of focus groups, and applicable to this young adult population. Focus groups promote exploration and brainstorming to discuss possible solutions. Responses from the focus groups are being used to inform the development of a young adult hypertension intervention.

Patients were recruited by the Wisconsin Research & Education Network (WREN), a statewide practice-based research network. Eligible patients were identified by electronic health record data, and were then mailed research study letters, on behalf of their clinic and primary providers, inviting them to participate in the study. Study recruitment flyers were also placed in the waiting areas of the selected Family Medicine Clinics, allowing patients to directly contact the research office if interested. Inclusion criteria included young adults, 18–39 years old, with an ambulatory ICD-9 hypertension diagnosis code [31] within the past 12 months of the study invitation, and awareness of their hypertension diagnosis (determined through telephone pre-screening). One focus group for each age group (18–29 year-olds and 30–39 year-olds) was conducted at each geographic site, for a total of 2 focus groups at each site. The recruitment goal was 6 individuals in each focus group to provide adequate variability in experiences to inform the development of a targeted young adult hypertension intervention [32–34]. Qualitative methods seek variation in perspectives and experiences to promote the emergence of themes [32, 35].

A semi-structured interview guide was developed based on previous literature on barriers to hypertension control [18, 19, 36, 37] and managing cardiovascular risk factors among adolescents/young adults (e.g., diabetes [38, 39], hypertension [25, 40, 41]). The focus group questions explored young adults’: 1) emotions and reactions after a hypertension diagnosis, 2) attitudes about managing hypertension (lifestyle changes, follow-up visits, antihypertensive medication use), 3) opinions about their healthcare system’s hypertension education materials, and 4) opinions about using social media (e.g., Facebook, Twitter, etc.) to manage hypertension. The development of these questions were guided by our prior research [24, 25] and input from healthcare system hypertension quality improvement committees. The focus group questions were reviewed, edited, and approved by the Community Advisors on Research Design and Strategies (CARDS^®^), a patient research advisory group coordinated by the Wisconsin Network for Research Support.

All focus groups were moderated, digitally audio recorded, and transcribed verbatim by the University of Wisconsin (UW) Survey Center. Prior to starting the focus group, all participants reviewed an IRB-approved summary sheet about the research study and provided verbal consent; written consent (signature) was waived by the University of Wisconsin-Madison Health Sciences Institutional Review Board. Each focus group was 90 min in length. Immediately after the focus group, each participant completed a written, anonymous, 5-question demographic survey (self-reported age, gender, race, ethnicity, and highest level of education achieved) and received a $75 cash honorarium. All transcriptions were reviewed for accuracy by the research staff (RW). All data was collected between June 2014 and December 2014.

### Data analysis

Following each focus group, data were analyzed by the research team, and subsequent interview questions were developed. Conventional content analysis was used to code the interview transcripts [42]. Initially, transcripts were read to achieve immersion and context. All codes were then determined from the transcribed text, rather than being generated *a priori*. Two investigators, without prior clinical hypertension experience (RW and JL) and from different disciplines (Rehabilitation Psychology; Education), independently coded all transcripts. Investigators without clinical hypertension knowledge were selected to avoid interpretation bias of patients’ experiences [43]. Emergent codes were generated in initial readings of the transcripts by each code. The coders then met bimonthly to review codes, adjudicate differences by consensus, and refine codes. When the final coding scheme was determined, following completion of all interviews, one coder (JL) recoded all transcripts using the final coding scheme. Data were managed by the coders with Excel. Methodological strategies undertaken to maintain rigor included the use of a multidisciplinary research team, member checking [44], and maintenance of memos [45].

## Results

Table 1 summarizes the demographics of each focus group by age group and Family Medicine clinic. All 38 young adults participated in focus groups and completed demographic questionnaires (mean: 26.7 [9.6] years old, 34 % male, 45 % Black, 42 % with ≥1 year of college).

**Table 1:** Baseline demographics of young adult focus groups respondents (18–39 years old)

	All Respondents (*n* = 38)	Academic clinic		Rural clinic		Urban clinic	
		18–29 year-olds (*n* = 6)	30–39 year-olds (*n* = 6)	18–29 year-olds (*n* = 6)	30–39 year-olds (*n* = 6)	18–29 year-olds (*n* = 7)	30–39 year-olds (*n* = 7)
Age, years, *m (SD)*	26.7 (9.6)	26.2 (1.8)	34.2 (3.1)	23.3 (3.2)	34.5 (2.6)	25.3 (2.7)	34.1 (1.7)
Male gender, *n (%)*	13 (34)	3 (50)	0 (0)	4 (67)	3 (50)	1 (14)	2 (29)
Race, *n (%)*							
White	20 (53)	3 (50)	4 (67)	6 (100)	6 (100)	1 (14)	0 (0)
Black	18 (47)	3 (50)	2 (33)	0 (0)	0 (0)	6 (86)	7 (100)
Other (including mixed race)	0 (0)	0 (0)	0 (0)	0 (0)	0 (0)	0 (0)	0 (0)
Ethnicity, *n (%)*							
Hispanic or Latino	0 (0)	0 (0)	0 (0)	0 (0)	0 (0)	0 (0)	0 (0)
Highest Level of Education, *n (%)*							
≤ 8th Grade	0 (0)	0 (0)	0 (0)	0 (0)	0 (0)	0 (0)	0 (0)
Some high school	3 (7.9)	0 (0)	0 (0)	1 (17)	0 (0)	1 (14)	1 (14)
Completed High School or GED equivalent^a^	17 (45)	3 (50)	3 (50)	2 (33)	3 (50)	3 (43)	3 (43)
Some college or vocational school	9 (24)	1 (17)	1 (17)	1 (17)	1 (17)	3 (43)	2 (29)
Completed college or vocational school	7 (18)	1 (17)	2 (33)	1 (17)	2 (33)	0 (0)	1 (14)
Graduate or Professional School	2 (5.3)	1 (17)	0 (0)	1 (17)	0 (0)	0 (0)	0 (0)

^a^ *GED* General Education Diploma

### Emotions and reactions after an initial hypertension diagnosis

The clinical term “high blood pressure”, instead of “hypertension”, was used during the focus groups. Previous research by Morgan and Watkins [26] demonstrated that many young adults do not understand the meaning of “hypertension” and/or cannot equate it to “high blood pressure”.

Young adults with hypertension were asked to think about when they were first diagnosed with “high blood pressure” (Table 2). All of the respondents, except one (37/38), provided at least one emotion; the most common emotions (30/37; 81 %) were “surprised”, “scared”, and “angry”. The one exception was a respondent that was not aware that hypertension (high blood pressure) is a chronic disease diagnosis.

**Table 2:** Example focus group questions

Emotions/Reactions After Initial Hypertension Diagnosis	Thinking back to when you first found out you have high blood pressure:
	a) What were some of your first thoughts or questions?
	b) What emotions did you feel?
Attitudes About Managing Hypertension	a) If your doctor recommended that you exercise to lower your blood pressure, what might keep you from exercising?
	b) If your doctor recommended that you eat healthier to lower your blood pressure, what might keep you from eating healthier?
	c) If your doctor recommended medication for your blood pressure, what would keep you from taking the medication?
	d) What would you do if you didn’t like your doctor’s plan or recommendation for treating your blood pressure?
Hypertension Education Materials	a) How many of you have received any information, such as handouts or printouts, from your clinic about exercise or healthy foods to help you lower your blood pressure?
	b) What might keep you from using the information you received from your clinic?
Social Media to Manage Hypertension	Would you use social media such as Facebook or Twitter to get support and tips about living with high blood pressure?

- 6005: “Scared. Because I heard so many things about it. If you don’t take care of it, you can really be messed up on it, like a stroke and heart attacks and stuff like that.”

Some young adults (7/38, 18 %) described an additional emotion of self-blame after their initial hypertension diagnosis; these 7 respondents identified a hypertension diagnosis as being their fault and a reflection of poor choices. However, 26 % (10/38) were concerned about negative stigma associated with hypertension; these 10 respondents were concerned about experiencing “shame” from their peers without hypertension or being stigmatized for being unhealthy. Although additional individuals did not voice similar responses, audiotapes captured significant group response of “yeah” or “I know” after quotes like the one below.

- 5006: “I think that high blood pressure is one of those conditions that is generally seen as being one’s own fault for getting it. So, if you have cancer, people are going to feel bad for you, you’ll get support. But high blood pressure, I think a lot of people have misconceptions about it and they wouldn’t feel bad for you or give you support. They might, probably not publicly but privately, say ‘They should just eat better or lose some weight, exercise. It’s their own fault.’”

Twenty-nine of the 38 young adult respondents (76 %) shared that they *expected* to develop hypertension during their lifetime. Among those 29 young adults, none of the respondents expected to develop hypertension at a young age. However, 20 of the 29 (69 %) had prior experience(s) with family members managing hypertension as a chronic disease and also experiencing complications of hypertension (e.g., stroke, heart attack, death).

- 1005: “I didn't expect it that early but I knew that at some point I would probably be diagnosed because it runs on both sides of my family.”

### Young adults’ knowledge about hypertension and future health risks

Overall, we noticed dichotomous responses to questions related to hypertension knowledge and the delivery of hypertension education (diagnosis, treatment, chronic management, and complications). For hypertension knowledge, all (100 %) of the young adults verbally gave at least one health complication associated with hypertension (e.g., heart attack, stroke, death). This likely reflects the prevalence of a family history of hypertension and its complications discussed above. However, across both age ranges of focus groups, none of the young adults were aware of chronic kidney disease as a complication of hypertension, despite the increased prevalence of chronic kidney disease, even among young adults in the U.S. [46].

Less than half of the respondents (*n* = 16, 42 %) reported receiving hypertension education (e.g., counseling, written materials, etc.) from their clinic and/or healthcare team. Among the respondents that reported receiving handouts/pamphlets from their clinic, across clinics and geographic sites, >50 % reported discarding the materials prior to leaving the clinic. The most common reasons for discarding materials included: 1) lack of self-efficacy (not believing they were able to accomplish the recommended behavior changes), 2) information redundancy, and 3) the information did not address their specific questions.

- 2004: “…so they gave me a whole bunch of pamphlets. I left them on the side of like the waiting chair. You know they just try to tell you you’re just going to have to do this the rest of your life pretty much, unless you drastically lose 100 lb and stop smoking a pack of cigarettes and just right now I can’t do that.”

Twenty-one respondents (55 %) shared that their primary hypertension knowledge source was family and/or their own internet site search. Respondents most frequently inquired about blood pressure medication with family members; they were felt to be more of a trustworthy source than their healthcare team. In addition, young adults believed that they were more likely to tolerate a prescription medication if their family member was also on the same medication. Other respondents denied obtaining any information from their healthcare team or independently.

- 2006: “I wasn’t given any sort of information about it really. She eventually diagnosed me and gave me like half of a pill to take. I wasn’t like you know told to make any particular like diet changes or anything like that. It was just like oh, you have this, ok.”

### Young adults’ attitudes and barriers to hypertension lifestyle modifications

Many young adults felt that guideline-recommended lifestyle modifications to manage hypertension equated to them no longer enjoying life and required them to live as if they were older than their biological age. All (100 %) of the young adults that were knowledgeable about lower sodium to treat hypertension felt they were unable to achieve the lower sodium goal (1500–2000 mg/day). Across all audiotapes, lower sodium (i.e., lower salt) was interpreted as lack of enjoyment with food.

- 6003: “I can’t eat no more, like he was saying, tacos, cheese steaks…. You know what I’m talking about? Like I’m only 34 but I got to live like I’m 67 and some s***. I don’t like that.”

Approximately 30 % (*n* = 12) felt that achieving recommended physical activity was “easier” than achieving dietary health behavior goals. For the majority of the 12 respondents, reasons included physical activity already being integrated into their life (e.g., work, children, family). For the remaining, they were more confident about starting a new physical activity health behavior because it is a more independent change and not influenced as much by social circumstances and peer influences. In contrast, having a healthier plate/meal option may promote inquiries by family and friends.

All of the respondents across both ages of focus groups commented on the cost of healthy food options compared to higher sodium and/or higher fat food options when grocery shopping. Their responses highlighted that they had personally investigated healthier food options because they were able to give comparisons for specific products (e.g., chicken breast vs. legs; fresh vs. canned vegetables). In addition, one respondent commented on how quickly fresh vegetables spoil prior to consumption and the effect on her food budget.

- 6002: “I want to go buy us some meats and stuff and I ain’t going to be thinking about no vegetables, because vegetables spoil fast. I open up the lettuce, I have to use that lettuce within 3 days. So my lettuce done went to waste and I done spent two, three dollars on some lettuce.”

One young male discussed difficulty selecting fast-food options when traveling for work. Not only did he have difficulty selecting a healthier option, but he shared experiences about fast food employees’ negative reactions when he ordered a healthy item (e.g., salad). Throughout our focus groups, our respondents had a recurring theme of societal and peer responses when a healthy choice does not fit the perceived “young adult” norm.

- 1005: “There’s no healthy food options for quick food when you’re traveling. It’s like at burger joints people will look at you cross eyed if you want to order a salad. ‘Are you really going to trust our salad?’”

Time was a much more common barrier in our 30–39 year-old focus groups compared to our 18–29 year-old groups. All of the women in the older group discussed trying to balance being a new mom with other responsibilities, with the most common being work and spending time with their significant other. Two mothers also discussed trying to balance a full-time job with school part-time. The combined responsibilities made it difficult for young adults to make time for healthy behavior changes (e.g., meal planning, preparing different meals, home cooked meals, exercising).

- 1002: “Being a new mom too. It’s tough to balance work and spending time with the baby and spending time with my spouse. It’s hard to balance all that, and then rationalize, like well I could go, you know, by myself and workout but I haven’t seen anybody all day, so I’d rather just play on the floor here.”

### Cost-benefit analysis of adhering to blood pressure medication

Multiple themes emerged about young adults weighing the risks and benefits of high blood pressure medication adherence. The most common recurring theme 31 % (12/38) was that hypertension is commonly asymptomatic. There are usually no negative health repercussions on a day-to-day basis for skipping blood pressure medication. Of these 12 respondents, 6 shared that the complications of hypertension are usually long-term, which promotes a young adult population to take more of a risk.

- 5006: “I think, specifically with things like high blood pressure, you can’t tell if you missed a pill. It’s not like, ‘l missed my pill today but oh, this is going to happen.’ It’s such a long-term thing…don’t take it that seriously if I miss one day. It’s a long-term thing. But then, one day. One day. A week. It all adds up.”

Overall, approximately 55 % (21/38) of young adults were prescribed antihypertensive medication(s). The majority of these young adults felt that medication cost was no longer a barrier to blood pressure medication, with the primary reason being the United States’ Affordable Care Act [47, 48]. Over 30 % of the 21 young adults shared stories of financial barriers prior to obtaining prescription insurance. Remembering to take medication(s) and side effects were the most common active barriers across all ages of our focus groups. Although our moderators did not ask, some young adults volunteered methods that they found effective to remember medications (e.g., take when brushing teeth, put next to morning coffee, set alarm, etc.) within our interactive focus groups.

Medication side effects was a concern among all 21 young adults prescribed blood pressure medication. Respondents shared concerns about long-term side effects. Also, since many had experience with hypertension through other family members, there was concern about being prescribed medication that another family member may not have tolerated. Lastly, approximately 5 patients (13 %) did not believe they needed medication and instead felt that stress management would be the most effective treatment. All of the themes about medication non-adherence merged back to the cost-benefit analysis. Young adult respondents shared that many times patients will gamble when immediate negative repercussions are not expected.

- 6001: “I think most people just gamble. You know? I mean, when I say a gamble, is that you figure ain’t nothing going to happen to you until it happens. And once it happens to you, you say ‘I’m gonna, I wish I would of’ and sometimes it’s too late, because you dead.”

### Social media use among young adults for hypertension self-management

The latter part of our focus group addressed possible social media solutions to improve hypertension education, self-management, and control among young adults. Contrary to our hypothesis, the majority of respondents disliked any social media (e.g., Facebook, Twitter, etc.) or text messaging communication for hypertension self-management. Young adults could not recommend a preferred alternative social media platform. They were concerned about peers finding out about their hypertension diagnosis. Some young adults felt that it would be an invasion of their personal space or result in harassment. In addition, for many respondents, social media was a place to avoid life stressors and they did not want their healthcare issues overlapping with recreational activities. Both ages preferred readings directly from internet websites – which allowed privacy, portability, and flexibility with their schedules.

## Discussion

This is the first multi-center qualitative study to assess barriers to hypertension control among U.S. young adults (18–39 year-olds) with regular primary care access. Our research adds to current knowledge gaps by addressing both behavior modification and pharmacologic treatment for hypertension. In contrast to prior hypertension qualitative studies in other age groups, our young adult focus group respondents highlighted a change in self-identity upon receiving a diagnosis of hypertension. This chronic disease diagnosis and the recommended lifestyle modifications cause many young adults to feel “older” than their biological age. Our focus groups respondents also shared ongoing adverse psychological effects of having a hypertension diagnosis, including self-blame and shame. These findings help fill an important research gap in understanding young adults’ experiences as identified by Marshall, et al [30]. We have highlighted the need for healthcare teams to understand the negative emotional and mental health effects a hypertension diagnosis has on young adults [49, 50]. Similar results have been obtained for other chronic diseases (e.g., asthma [50], diabetes); however, additional research and resources are needed to address this critical barrier, specifically among young adults.

Another important emergent theme was the cost-benefit analysis performed by young adults when determining the necessity of a recommended blood pressure treatment plan (e.g., lifestyle modifications, medication). This implicit cost-benefit analysis has been demonstrated among other chronic diseases and has been shown to be associated with medication adherence [51]. We have demonstrated that this cost-benefit analysis is an important barrier to hypertension control in young adults. Our U.S. data supports prior qualitative studies, which demonstrated that medication non-adherence is influenced by concerns about long-term harmful effects [52], and difficulty balancing other life responsibilities with finding time to take medication and follow-up with their healthcare team [30]. These findings also support prior research demonstrating a significant decrease in clinic visit attendance during transition to young adulthood [53] and young adults’ goal of having treatment plans comply with their lifestyle, not with their doctor’s wishes [54].

We also demonstrated that young adults perform a cost-benefit analysis when selecting healthy food options. Young adults are a unique population, usually with newer jobs and, for many, new budgeting responsibilities. Their observation of the higher cost of many healthy foods in the U.S. compared to higher sodium foods is a critical barrier to health behavior adoption. These findings highlight the need for ongoing policy changes. Finally, young adults’ concerns about experiencing negative social stigma based upon their behavior choices, especially among males, results in a cost-benefit analysis for behavior modification (e.g., ordering high sodium foods, rather than a salad). Unfortunately, young adults shared that negative stigma in response to behavior change expands beyond their daily activities in society and also effects their behaviors and choices at home among family. Effective, team-based young adult-healthcare team communication is needed to discuss patients’ beliefs and concerns, and jointly develop a treatment plan that individually addresses other competing demands in young adulthood.

Our research also underscores the need for hypertension education materials tailored specifically to young adults’ questions and concerns. Similar to prior findings by other researchers [29, 52], our young adult respondents were aware of the importance of controlling their blood pressure, with many having unfortunate cardiovascular events in their family. However, additional research is needed to improve the *delivery* of hypertension education materials to young adults to promote necessary behavioral changes to achieve and maintain hypertension control. Many young adults reported discarding hypertension education materials prior to leaving the clinic. Since providers use printed education materials to supplement limited counseling time, discarded materials result in young adults receiving suboptimal education about hypertension as a diagnosis, its contributors, and ongoing chronic disease management. We also identified that overall, current hypertension materials are not beneficial in helping young adults initiate or maintain lifestyle modifications. Broad healthcare system changes are needed to improve the delivery of tailored hypertension education materials and integrate effective resources to support the maintenance of lifestyle modifications. According to previous studies, behavior change is more likely to be maintained when lifestyle changes are integrated into one’s identity and are internally motivated [55, 56], key factors lacking in the current delivery of hypertension education to young adults. Some of our identified hypertension control barriers (e.g., co-payments, medication side effects) overlapped with previous studies in other age groups [19, 30, 36], but demonstrate ongoing needs to improve hypertension control across populations.

In contrast to our hypothesis, young adults did not like social media or text messaging to assist with hypertension chronic disease management. These mobile health options were considered an intrusion on their recreational activities and increased their concerns of peers being aware of their diagnosis. Social networks using group interactions in middle-aged and older adults have been beneficial in managing hypertension [57]. However, further research is needed to understand the support resources that meet the needs and protect the self-identity of young adults.

In summary, our findings expand prior research in hypertension treatment adherence by studying both medication and lifestyle modification adherence. Prior frameworks focused on adherence [58, 59] and often addressed patients’ health beliefs about an illness. However, we demonstrated that among 18–39 year-olds, patients’ belief in their own self-identity as a “young” adult is negatively altered. Additionally, solely focusing on social support outside of the clinic may not be a successful intervention, since many young adults often consider hypertension a “private” issue among their peers and even among family members without hypertension. These findings highlight the need for expanded team-based care to increase patient-provider communication and to increase perceived autonomy support and self-management resources for young adults with hypertension [60, 61].

Strengths of this qualitative analysis include a multisite design of academic, rural, and urban healthcare systems, which represents a diverse sample of young adults with hypertension. Although young adults were from the Midwestern U.S., clinics across diverse geographic regions were represented. Overall, 45 % of the focus group respondents were Black; however, there was a lack of Latino representation. Therefore, this data may not encompass some barriers encountered in managing hypertension across other minority races/ethnicities. Our focus group design may not have allowed respondents to provide in-depth answers to all of their individual experiences with hypertension [62]. Yet, our goal was to foster interactions between young adults to move the discussion beyond individual experiences and brainstorm about possible interventions for young adults. There is always a concern that some participants do not actively participate in group discussions. However, our trained moderators from the University of Wisconsin Survey Center provided an environment for each individual to participate with every question, as reviewed on the audiotape files, and to reduce the likelihood of solely dominant personalities responding. Additionally, there is a concern that young adults may be uneasy discussing a chronic medical issue in public. As expected, almost 30 % of the respondents shared that a diagnosis of hypertension is a private issue when around peers or family *without* hypertension. To address this concern *a priori*, we ensured that each focus group was held within the patients’ normal primary care clinic in a closed conference room. Finally, all young adults in this study had regular primary care access (i.e., a medical home) and the identified barriers in this manuscript may have a different priority and/or may not reflect young adults without primary care access. However, these results will help inform the adaptation of effective hypertension interventions to young adult populations and likely improve the low and stagnant hypertension control rates in this population.

## Conclusions

This qualitative analysis identifies important intervention target areas for young adults with hypertension. Developing interventions to address young adults’ unique needs are important next steps to improve hypertension control and cardiovascular preventive healthcare delivery.

## Abbreviations

CARDS: community advisors on research design and strategies
IRB: institutional review board
UW: University of Wisconsin
WREN: Wisconsin research and education network
